# Trends in the prevalence of hypovitaminosis D over a 10-year period in Japan: the research on osteoarthritis/osteoporosis against disability study 2005–2015

**DOI:** 10.1007/s11657-025-01601-9

**Published:** 2025-08-27

**Authors:** Noriko Yoshimura, Toshiko Iidaka, Chiaki Horii, Gaku Tanegashima, Shigeyuki Muraki, Hiroyuki Oka, Hiroshi Kawaguchi, Toru Akune, Kozo Nakamura, Sakae Tanaka

**Affiliations:** 1 Department of Prevention Medicine for Locomotive Organ Disorders, 22th Century Medical and Research Center, The University of Tokyo, Tokyo, 113-8655 Japan; 2 Department of Orthopaedic Surgery, Sensory and Motor System Medicine, Graduate School of Medicine, The University of Tokyo, Tokyo, 113-8655 Japan; 3 Department of Medical Research and Management for Musculoskeletal Pain, 22nd Century Medical and Research Center, The University of Tokyo, Tokyo, 113-8655 Japan; 4 Nodogaya Hospital, Chiba, 277-0084 Japan; 5 National Rehabilitation Center for Persons With Disabilities, Saitama, 359-0042 Japan; 6 Towa Hospital, Tokyo, 120-0003 Japan

**Keywords:** Vitamin D deficiency, Vitamin D insufficiency, Prevalence, Secular trend, Population-based cohort ROAD study

## Abstract

**Summary:**

We examined the trends in vitamin D insufficiency and deficiency over a 10-year period in the general population. The prevalence of deficiency significantly decreased (29.5% vs. 21.6%), whereas mean serum levels increased (23.3 ng/mL vs. 25.1 ng/mL). These trends may reduce the incidence of osteoporosis and osteoporotic fractures.

**Purpose:**

We aimed to clarify the trends in the prevalence of vitamin D insufficiency and deficiency in the general population using population-based cohort data from a baseline survey and a follow-up survey conducted 10 years later.

**Methods:**

A baseline survey of the Research on Osteoarthritis/Osteoporosis Against Disability (ROAD) study was conducted from 2005 to 2007. Blood samples were collected to measure serum 25-hydroxyvitamin D (25D) and intact parathyroid hormone levels from 1,683 participants (595 men, 1,088 women). Participants also completed an interviewer-administered questionnaire, and underwent bone mineral density measurements and radiographic examinations. The fourth survey was conducted from 2015 to 2016 with 1,906 individuals (637 men, 1,269 women), including both follow-up participants from the baseline survey and newly recruited individuals to increase the sample size for future longitudinal analyses. All participants completed assessments identical to those in the baseline survey. Vitamin D deficiency and insufficiency were defined as serum 25D levels of < 20 ng/mL and ≥ 20 ng/mL but < 30 ng/mL, respectively.

**Results:**

The mean serum vitamin D levels were 23.3 ng/mL at baseline and 25.1 ng/mL at the fourth survey, indicating a significant increase (*p* < 0.001). The prevalences of vitamin D insufficiency and deficiency were 52.9% and 29.5%, respectively, at baseline, and 54.8% and 21.6%, respectively, in the fourth survey, indicating a significant decrease in vitamin D deficiency (*p* < 0.001).

**Conclusions:**

In this population-based survey with a 10-year interval, the prevalence of hypovitaminosis D significantly decreased. This favourable trend may contribute to future reductions in the incidence of osteoporosis and osteoporotic fractures.

## Introduction

Vitamin D plays a crucial role in bone health, particularly in maintaining bone mineral density (BMD) [1, 2]. Deficiency in vitamin D can lead to decreased bone mineralisation, secondary hyperparathyroidism, and increased cortical bone loss, all of which contribute to the pathogenesis of osteoporosis and hip fractures [1, 3].

However, vitamin D status significantly varies across populations. A comprehensive review by Mithal et al. examined population-based reports on vitamin D status across six global regions (Asia, Europe, the Middle East and Africa, Latin America, North America, and Oceania) and found that serum 25-hydroxyvitamin D (25D) levels < 75 nmol/L (< 30 ng/mL) were prevalent in all regions, with levels < 25 nmol/L (< 10 ng/mL), particularly in South Asia and the Middle East [4]. Furthermore, the International Osteoporosis Foundation (IOF) reported that vitamin D deficiency (25D levels < 30 ng/mL) was alarmingly high among postmenopausal women, with prevalences of approximately 50% in Thailand and Malaysia, 75% in the United States, and 90% in Eastern Asia [5].

In Japan, an Eastern Asian country, the prevalence of vitamin D inadequacy among postmenopausal women is high [5]. Nakamura et al. measured the serum 25D levels in 600 postmenopausal women and found a significant association between higher 25D concentrations and higher femoral neck BMD. Their study indicated that at least 20 ng/mL 25D was necessary to achieve normal parathyroid hormone (PTH) levels and prevent low BMD [6]. In addition, our previous study on the Japanese community-dwelling population, which utilised data from a population-based cohort known as the Research on Osteoarthritis/Osteoporosis Against Disability (ROAD), highlighted the high prevalence of 25D insufficiency and deficiency. The baseline survey conducted between 2005 and 2007, involving 1,683 individuals, revealed that the overall prevalences of vitamin D insufficiency and deficiency were 81.3% and 1.2%, respectively, using the following criteria: vitamin D insufficiency, 25D serum levels ≥ 10 ng/mL and < 30 ng/mL; and vitamin D deficiency, 25D serum levels < 10 ng/mL. The prevalence was notably higher in women than in men (vitamin D insufficiency: men 72.1%, women 86.3%; vitamin D deficiency: men 0.3%, women 1.7%) (*p* < 0.001) [7–9].

Following the baseline survey of the ROAD study, identical surveys were conducted in the same communities in 2008–2009, 2012–2013, 2015–2016, 2018–2019, and 2022–2023. During the second (2008–2009), fourth (2015–2016), and fifth (2018–2019) follow-up surveys, serum 25D, intact PTH (iPTH), calcium (Ca), and phosphate (P) levels were remeasured. In the present study, we utilised data from the fourth survey (2015–2016), a 10-year follow-up from baseline, to analyse the age- and sex-stratified results of 25D, iPTH, Ca and P measurements.

The present study aimed to estimate and compare the prevalence of vitamin D insufficiency and deficiency with that of a baseline survey, with the goal of elucidating the 10-year trend in hypovitaminosis.

## Methods

### Study population

The ROAD study, initiated in 2005, is a national prospective study on musculoskeletal diseases, consisting of cohorts from several communities in Japan. Demographic details of the cohorts have been reported previously [7, 8]. Briefly, between 2005 and 2007, a baseline database was established, including clinical and genetic information from 3,040 residents (1,061 men and 1,979 women), with a mean age of 70.3 years (standard deviation [SD], 11.0) (71.0 [10.7] years for men and 69.9 [11.2] years for women). Participants were recruited from resident registration records of three communities with distinct characteristics. The first community, Itabashi, Tokyo, is an urban region; 1,350 participants (aged ≥ 60 years) were recruited from this community. The second community, Hidakagawa, Wakayama, is a mountainous region from which 864 participants (aged ≥ 40 years) were enrolled. The third community, Taiji, Wakayama, is a coastal region from which 826 participants (aged ≥ 40 years) were recruited.

Of the 3,040 participants, 1,690 individuals (596 men and 1,094 women) with a mean age of 65.2 years (SD, 12.0) (66.3 [11.7] years for men and 64.7 [12.1] years for women) who participated in the ROAD study and were recruited from the mountainous and coastal regions were analysed. Participants from urban regions were excluded because baseline BMD measurements using dual-energy X-ray absorptiometry (DXA) were not performed. Blood and urine examinations were conducted only in participants from the mountainous and coastal regions. Among the 1,690 participants, serum 25D levels were measured in 1,683 individuals (99.6%; 595 men, 1,088 women), and data from these 1,683 participants were used for the analysis in the present study.

The fourth ROAD study, conducted in 2015–2016, was implemented in the same mountainous and coastal communities as those included in the baseline survey, ensuring regional consistency. This study served as a 10-year follow-up of the baseline survey and provided a new baseline for the subsequent 10 years. As in the original study, individuals were invited to participate through publications on town hall public relations, and residents aged > 40 years were encouraged to participate. Additionally, individuals who had participated in previous ROAD surveys (baseline, second, and third surveys) were invited via letters. Similar to the baseline study, individuals aged < 40 years who wished to participate were included. The inclusion criteria for the baseline study were as follows: participants were able to (1) visit the clinic where the survey was conducted, (2) provide self-reported data, and (3) understand and sign an informed consent form. No other exclusion criteria were applied.

To increase the sample size and improve the representativeness of the cohort for future follow-up, the fourth survey included both individuals who continued their participation from earlier phases of the study and newly recruited participants. Of the 1,683 individuals in the baseline survey, 1,119 (66.5%; 373 men and 746 women) also took part in the fourth survey as part of longitudinal follow-up. Including the new recruits, the total number of participants in the fourth survey was 1,906 (636 men and 1,270 women), with a mean age of 65.0 years (SD, 12.7)—65.1 (13.3) years for men and 64.9 (12.4) years for women. Among the 1,906 participants, serum 25D levels were measured in 1,902 individuals (99.8%; 636 men, 1,266 women), and data from the 1,902 participants in the fourth survey were used for the analysis of comparison to the baseline in the present study.

Informed consent was obtained from all participants, and this study was approved by the Ethics Committees of the University of Tokyo (Nos. 1264 and 1326) and Wakayama Medical University (No. 373).

### Baseline and fourth surveys of the ROAD study

#### Interviewer-administered questionnaire

The participants completed an interviewer-administered questionnaire covering lifestyle factors, including occupation, smoking habits, alcohol consumption, family history, past/present medical information, medication use, physical activity, reproductive history, and health-related quality of life.

#### Anthropometric measurements and medical examination

Anthropometric measurements, including height and weight, were taken for all participants, and body mass index (BMI) was calculated as weight (kg)/height (m^2^). The medical information of all participants was collected by experienced orthopaedic surgeons.

#### Blood and urine examinations

Baseline samples were collected between October and January in both the mountainous and coastal areas. The samples for the fourth survey were collected between October and December in both regions. Blood and urine samples were collected between 09:00 and 15:00. After centrifugation, serum and urine samples were immediately placed on dry ice and transferred to a deep freezer within 24 h. These samples were stored at –80 °C until analysis.

Using serum samples, serum 25D and iPTH levels were measured. Additionally, levels of Ca and P were determined. Serum 25D was quantified using a radioimmunoassay with an 125I-labelled tracer (DiaSorin, Stillwater, MN, USA) [10], and iPTH was analysed using an electrochemiluminescence immunoassay (Roche Diagnostics GmbH, Mannheim, Germany). Serum Ca and P were measured by an external laboratory using standard clinical chemistry methods—o-Cresolphthalein Complexone (CPC) method for Ca, and the molybdenum method for P.

#### Bone mineral density measurement

BMD was measured at the lumbar spine (L2–4) and proximal femur using dual-energy X-ray absorptiometry (DXA) with the Hologic QDR Discovery system (Hologic, Waltham, MA, USA) in both the baseline and fourth surveys. To ensure consistency, the same DXA machine was used across both regions and survey periods, and a standard spine phantom was scanned daily to monitor machine performance. The phantom BMD was maintained at 1.032 ± 0.016 g/cm^2^ (± 1.5%) throughout all measurements. Additionally, all participants were examined by a single physician (N.Y.) to minimize inter-observer variability. The coefficient of variation (CV) for lumbar spine BMD (L2–4) measured using the Hologic QDR Discovery system has been reported to be 1.0%, while that for femoral neck BMD is 1.5%, indicating high measurement precision [11].

### Definition of osteoporosis

Osteoporosis was defined according to the World Health Organisation (WHO) criteria [12, 13]. The mean L2–4 BMD in young adult men and women as measured by hologic DXA in Japan is 1.011 (SD, 0.119) g/cm^2^ [14]. Osteoporosis of the lumbar spine was defined as L2–4 BMD < 0.714 g/cm. The mean BMD values of the femoral neck in young adult men and women were 0.863 (SD, 0.127) and 0.787 (SD, 0.109) g/cm^2^, respectively, and the osteoporosis of the femoral neck was defined as a BMD < 0.546 g/cm^2^ in men and < 0.515 g/cm^2^ in women [14].

### Definitions of vitamin D insufficiency and deficiency

To assess the severity of vitamin D status worldwide, the IOF reported vitamin D status using four categories based on mean (or median) 25D levels: ≥ 30 ng/mL, 20–30 ng/mL, 10–20 ng/mL, and < 10 ng/mL [15]. In Japan, an expert panel supported by the Research Program of Intractable Diseases; Ministry of Health, Labour and Welfare; Japanese Society for Bone and Mineral Research, and Japan Endocrine Society issued an opinion in 2017 stating that vitamin D deficiency should be defined as 25D serum levels < 20 ng/mL and vitamin D insufficiency as 25D serum levels ≥ 20 ng/mL and < 30 ng/mL [16]. Subsequently, the criterion for vitamin D deficiency in Japan’s health insurance system was set at < 20 ng/mL Therefore, in the present study, we adopted the Japanese definition, defining vitamin D deficiency as 25D serum levels < 20 ng/mL and vitamin D insufficiency as 25D serum levels ≥ 20 ng/mL and < 30 ng/mL.

### Statistical analyses

All statistical analyses were performed using the STATA statistical software (STATA Corp., College Station, Texas, USA). Differences in proportions were compared using the chi-squared test. Continuous variables were compared using analysis of variance for multiple groups or Scheffé’s least significant difference test for pairwise comparisons.

Multinomial logistic regression analysis was used to evaluate differences in the prevalence of vitamin D insufficiency and deficiency between the baseline (2005–2007) and fourth (2015–2016) surveys. Vitamin D insufficiency and deficiency (vs. normal vitamin D status) were treated as outcome variables, and the survey round (baseline vs. fourth) as the explanatory variable. The model was adjusted for age, sex, BMI, residential area (mountainous or coastal), current smoking status, current drinking status, and season of blood sampling, categorized as autumn (October and November) or winter (December and January) based on the collection period. These variables were included in Model 1. In Model 2, additional adjustments were made for lumbar spine BMD (L2–4) and serum levels of iPTH, Ca, and P.

To identify factors associated with hypovitaminosis D in more recent years, we conducted a separate multinomial logistic regression analysis using data from the fourth survey only. As in the previous analysis, vitamin D insufficiency and deficiency (vs. normal vitamin D status) were used as outcome variables. The explanatory variables in Model 1 were the same as those used in Table 3—age, sex, BMI, residential area (mountainous or coastal), current smoking status, current drinking status, and season of blood sampling. However, unlike Table 3, the variable for survey round (baseline vs. fourth) was not included, since this analysis focused solely on the most recent time point. Model 2 additionally included lumbar spine BMD (L2–4) and serum levels of iPTH, Ca, and P.

## Results

### Study population

A total of 1,683 participants from the baseline study and 1,902 participants from the fourth survey were included. Table 1 shows the background characteristics of the participants at baseline and the fourth surveys. No significant differences were found in the age–sex distribution of the participants in the baseline and fourth surveys. However, the mean heights of both men and women in the fourth survey were higher than those in the baseline survey. Additionally, the mean weight of the men in the fourth survey was higher than that in the baseline survey, although no significant difference was observed in the mean weight of the women between the two surveys. Thus, the men in the fourth survey were generally larger than those in the baseline survey, whereas the women recruited in the fourth survey were comparatively slimmer. Moreover, the proportion of participants with a current smoking status was significantly lower in the fourth survey than in the baseline study, whereas the proportion of women with a current alcohol consumption history was significantly higher in the fourth survey than in the baseline survey.

**Table 1:** Comparison of background characteristics of the participants at the vitamin D measurements in the baseline survey (2005–2007) and those in the 4th survey (2015–2016)

	Total			Men			Women		
	Baseline	4th survey	*p*-value baseline vs. 4th survey	Baseline	4th survey	*p*-value baseline vs. 4th survey	Baseline	4th survey	*p*-value baseline vs. 4th survey
Number of subjects	1,683	1,902		595	636		1,088	1,266	
Selected characteristics (mean, (SD))									
Age (years)	65.3 (12.0)	64.9 (12.7)	0.4279	66.3 (11.7)	65.1 (13.3)	0.1044	64.7 (12.1)	64.9 (12.4)	0.7877
Height (cm)	155.2 (9.3)	157.3 (9.3)***	<0.0001	163.4 (7.2)	166.4 (6.9)***	<0.0001	150.7 (6.9)	152.7 (6.6)***	<0.0001
Weight (kg)	55.6 (10.8)	56.7 (11.6)**	0.0034	62.3 (10.9)	65.5 (11.4)***	<0.0001	52.0 (8.8)	52.4 (9.0)	0.3639
BMI (kg/m^2^)	23.0 (3.4)	22.8 (3.5)	0.0970	23.2 (3.2)	23.6 (3.4)	0.0721	22.9 (3.5)	22.4 (3.5)**	0.0020
Selected characteristics (%)									
Female sex	64,7	66,6	0.211	-	-	-	-	-	-
Measurement season (winter; December, January)	56.6	31.3***	<0.001	60.7	31.6***	<0.001	54.3	31.2***	<0.001
Residing in the coastal area	48.8	55.1***	<0.001	46.6	53.5*	0.015	50.1	55.9**	0.005
Current smoking habit, yes	13.1	9.4***	<0.001	29.8	21.7**	0.001	3.8	3.2	0.403
Current alcohol drinking, yes	39.7	42.8	0.061	66.7	69.2	0.345	25.0	29.5*	0.015
Mean values (SD) of BMD									
L2-4 (g/cm^2^)	0.93 (0.21)	0.97 (0.21)***	<0.0001	1.05 (0.20)	1.09 (0.21)***	0.0001	0.87 (0.18)	0.90 (0.17)***	<0.0001
Femoral neck (g/cm^2^)	0.67 (0.14)	0.66 (0.14)*	0.0423	0.75 (0.13)	0.76 (0.12)	0.1211	0.63 (0.12)	0.62 (0.11)***	0.0007
Prevalence of osteoporosis (%) #									
L2-4	13.6	9.8***	<0.001	3.4	1.4*	0.025	19.3	13.9**	0.001
Femoral neck	12.6	13.6	0.402	3.7	4.1	0.715	17.6	18.4	0.608

*SD* standard deviation, *BMI* body mass index, *BMD* bone mineral density, *L2-4* lumbar spine L2-L4

*p < 0.05, **p < 0.01, ***p < 0.001

# Osteoporosis is defined according to WHO criteria

Regarding BMD, both the lumbar spine L2–4 and femoral neck showed significantly higher values in the fourth survey than in the baseline survey (lumbar spine, *p* < 0.001; femoral neck, *p* < 0.05). When analysed based on sex, men demonstrated significantly higher BMD in the lumbar spine L2–4 in the fourth survey than in the baseline survey (*p* < 0.001), whereas no significant difference was observed in the femoral neck. In contrast, women had significantly higher BMD in both the lumbar spine and femoral neck in the fourth survey than in the baseline survey (*p* < 0.001) (Table 1).

Table 1 shows the prevalence of osteoporosis at baseline and in the fourth survey. The prevalence of osteoporosis in the L2–4 region in both men and women during the fourth survey was significantly lower than that at baseline (*p *< 0.01). However, there was no significant difference in the prevalence of osteoporosis of the femoral neck between men and women.

### Differences in serum 25-Hydroxyvitamin D, iPTH, Ca and P levels and prevalence of hypovitaminosis D between the baseline and fourth surveys

Table 2 shows a comparison of serum 25D, iPTH, Ca and P levels between the baseline and fourth surveys. The mean 25D levels in the fourth survey were significantly higher in both men and women than those in the baseline survey. In contrast, the mean iPTH values in the fourth survey were significantly lower in both men and women than those in the baseline survey. The mean serum Ca levels were 9.03 mg/dL (SD 0.47) in the baseline survey and 9.46 mg/dL (SD 0.37) in the fourth survey. The mean serum Pl evels were 3.53 mg/dL (SD 0.53) at baseline and 3.39 mg/dL (SD 0.52) in the fourth survey. Both differences were statistically significant (*p* < 0.0001),

**Table 2:** Comparison of values of serum 25-hydroxyvitamin D (25D),intact parathyroid hormone (iPTH), calsium (Ca) and phospate (P) in the baseline survey (2005–2007) and in the 4th survey (2015–2016)

	Total			Men			Women		
	Baseline	4th survey	*p*-value baseline vs. 4th survey	Baseline	4th survey	*p*-value baseline vs. 4th survey	Baseline	4th survey	*p*-value baseline vs. 4th survey
Number of subjects	1,683	1,902		595	636		1,088	1,266	
Mean values (SD) of serum items									
25D (ng/mL)	23.3 (6.6)	25.1 (7.0)***	<0.0001	25.7 (6.5)	28.6 (7.3)***	<0.0001	22.0 (6.2)	23.4(6.1)***	<0.0001
iPTH (pg/mL)	41.4 (33.9)	38.0 (17.6)***	0.0002	42.7 (52.0)	37.6 (19.2)*	0.0185	40.6 (17.4)	38.3 (16.8)***	0.0008
Ca (mg/dL)	9.03 (0.47)	9.45 (0.37) ***	<0.0001	8.86 (0.50)	9.41 (0.36) ***	<0.0001	9.09 (0.44)	9.48 (0.37) ***	<0.0001
P (mg/dL)	3.53 (0.53)	3.39 (0.52) ***	<0.0001	3.17 (0.53)	3.11 (0.51)	0.0831	3.65 (0.48)	3.53 (0.47)***	<0.0001
Prevalence of vitamin D status (%)									
Normal VD	17.5	23.6	<0.001	27.6	41.5	<0.001	12.0	14.5	<0.001
VD insufficiency #	52.9	54.8		55.5	49.2		51.6	57.7	
VD deficiency #	29.5	21.6		17.0	9.3		36.4	27.8	

*SD* standard deviation, *25D* 25-hydroxyvitamin D, *iPTH* intact parathyroid hormone, *Ca* calcium, *P* phosphate, *VD* vitamin D

*p < 0.05, **p < 0.01, ***p < 0.001

# Hypovitaminosis D is defined according to following Japanese criteria, vitamin D deficiency, 25D serum levels <20 ng/m, vitamin D insufficiency, 25D serum levels ≥20 ng/mL and <30 ng/mL

Table 2 also presents the prevalence of vitamin D insufficiency and deficiency in the baseline and fourth surveys. Compared with the baseline survey, the proportion of individuals with normal vitamin D levels (25D ≥ 30 ng/mL) was higher in the fourth survey than in the baseline survey, whereas the prevalence of vitamin D deficiency was lower. This trend was consistent for both men and women.

**Fig. 1:**
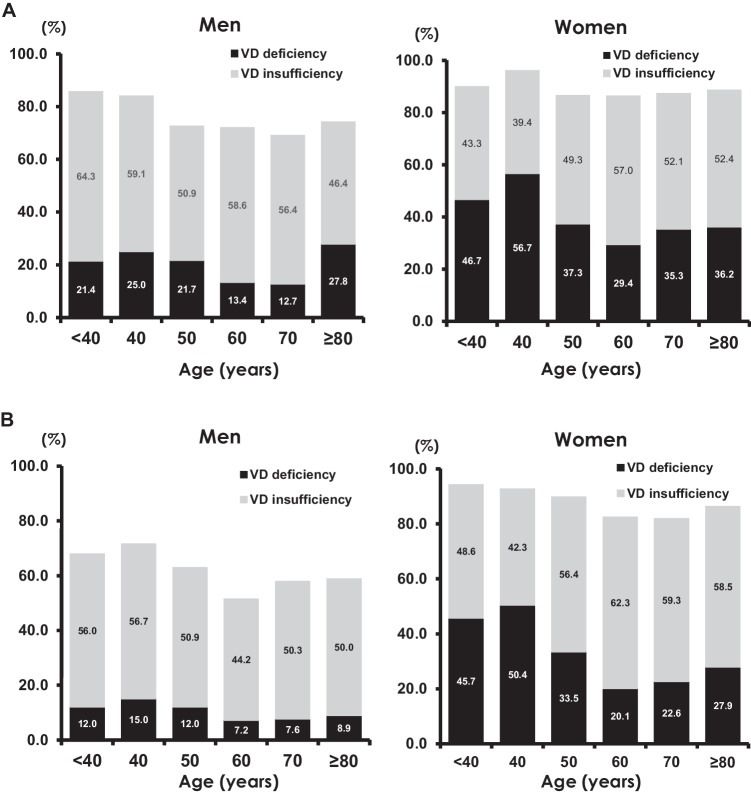
**A** Prevalence of hypovitaminosis D at the baseline survey. **B **Prevalence of hypovitaminosis D at the fourth survey

Figure 1 illustrates the prevalence of hypovitaminosis D in the baseline participants and those in the fourth survey, stratified based on sex and age (Fig. 1). Figure 1-a presents the results from the baseline survey, whereas Fig. 1-b presents the results from the fourth survey conducted 10 years later. This figure indicates that, in both the baseline and fourth surveys, the prevalence of vitamin D insufficiency and deficiency was significantly higher among women than among men (*p* < 0.001). Although the relationship with age is not clearly delineated in this figure, a notable reduction in the frequency of vitamin D deficiency was observed after 60 years of age.

**Fig. 2:**
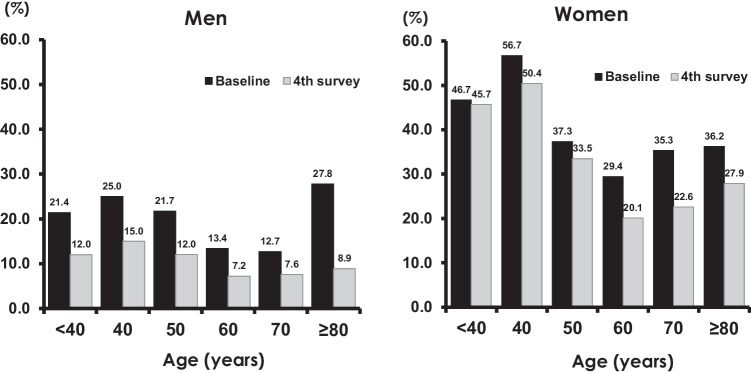
Comparison of vitamin D deficiency at the baseline and fourth surveys

To clarify the trend in the prevalence of hypovitaminosis D over this 10-year period, we isolated data for vitamin D deficiency and compared its prevalence between the baseline and fourth survey according to sex and age group (Fig. 2). This analysis revealed that, for both men and women, the frequency of vitamin D deficiency decreased in recent years (2015–2016) across all age groups.

To further investigate the trends in vitamin D insufficiency and deficiency over the 10-year period, we conducted a multinomial logistic regression analysis treating vitamin D status (normal, insufficiency, and deficiency) as the outcome variable and survey round (baseline or fourth) as the explanatory variable. Initially, without adjusting for any covariates, the Relative Risk Ratio (RRR) for vitamin D insufficiency was 0.92 (0.87–0.97) (*p* < 0.01) and that for vitamin D deficiency was 0.82 (0.76–0.87) (*p* < 0.001), indicating a significantly lower risk in the fourth survey compared with the baseline survey. Table 3 presents the results of the multinomial logistic regression analysis, in which vitamin D insufficiency and deficiency (vs. normal vitamin D) were treated as outcome variables, and survey round (baseline vs. fourth) as the main explanatory variable. In Model 1, the analysis was adjusted for age, sex, BMI, residential area, smoking and drinking habits, and season of blood sampling. In Model 2, additional adjustments were made for lumbar spine BMD (L2–4), serum iPTH, Ca, and P levels.

**Table 3:** Relative risk ratios (RRRs) for vitamin D insufficiency and deficiency, compared with normal vitamin D status, at the baseline (2005–2007) and fourth (2015–2016) surveys, adjusted for potentially associated factors

	VD insufficiency		VD deficiency	
Outcome: VD status (normal 0, insufficiency 1, deficiency 2)	Model 1 Adjusted RRR (95% CI)	Model 2 Adjutsted RRR (95% CI)	Model 1 Adjusted RRR (95% CI)	Model 2 Adjutsted RRR (95% CI)
The survey diagnosed with hypovitaminosis D (baseline 0, 4^th^ survey 1)	0.97 (0.91-1.04)	1.07 (0.99-1.15)	0.87 (0.81-0.93)***	1.01 (0.93-1.11)
Adjustment factors				
Sex (men 0, women1)	2.84 (2.31-3,50)***	2.98 (2.31-3,85)***	7.32 (5.58-9.62)***	10.9 (7.74-15.4)***
Age (+1 years)	0.988 (0.980-0.996)**	0.983 (0.976-0.992)***	0.967 (0.958-0.976)***	0.958 (0.949-0.968)***
BMI (+1kg/m^2^)	1.03 (1.00-1.06)*	1.04 (1.01-1.07)**	1.03 (0.997-1.06)	1.03 (0.99-1.07)
Residential area (0: mountainous area, 1: coastal area)	0.64 (0.53-0.77)***	0.61(0.50-0.74)***	0.44 (0.35-0.55)***	0.41 (0.32-0.53)***
Season of blood sampling (0: autumn, 1: winter)	2.13 (1.74-2.60)***	2.05(1.65-2.54)***	2.46 (1.94-3.11)***	2.27 (1.77-2.92)***
Current smoking habit, yes vs no	1.23 (0.93-1.64)	1.28 (0.94-1.75)	1.93 (1.35-2.76)***	2.27 (1.53-3.36)***
Current alcohol drinking, yes vs no	0.72 (0.59-0.88)**	0.73 (0.59-0.91)**	0.57 (0.45-0.72)***	0.45 (0.45-0.74)***
Additional adjustment factors				
BMD at L2-4 (+1 g/cm^2^)		0.60 (0.35-1.01)		0.74 (0.38-1.44)
Serum values of iPTH (+1 pg/mL)		1.01 (1.00-1.02)**		1.03 (1.02-1.03)***
Serum values of Ca (+1 mg/dL)		0.62 (0.49-0.79)***		0.37 (0.28-0.50)***
Serum values of P (+1 mg/dL)		1.01 (0.83-1.23		0.77 (0.61-0.98)*

*VD* vitamin D, *RRR* ralative risk ratio, *95% CI* 95% confidence interval, *BMI* body mass index, *BMD* bone mineral density, *L2-4* lumbar spine L2-L4, *iPTH* intact parathyroid hormone, *Ca* calcium, *P* phosphate

*p < 0.05, **p < 0.01, ***p < 0.001

In Model 1, after adjusting for demographic and lifestyle factors including age, sex, BMI, residential area, smoking and drinking habits, and season of blood sampling, the fourth survey still showed a significantly lower risk of both vitamin D insufficiency and deficiency compared with the baseline survey. However, when additional adjustments were made in Model 2 for physiological factors such as lumbar spine BMD (L2–4), serum iPTH, Ca, and P levels, the previously observed significant associations were attenuated and no longer statistically significant. This suggests that these variables may partly account for the decreased prevalence of hypovitaminosis D in the more recent survey.

### Factors associated with hypovitaminosis D in the fourth survey

To identify the factors associated with hypovitaminosis D, we analyzed data from the fourth survey. Table 4 presents the results of a multinomial logistic regression analysis. In Model 1, demographic factors such as sex and BMI, lifestyle habits (smoking and drinking), and environmental factors (season of blood sampling and residential area) were significantly associated with hypovitaminosis D. In Model 2, after additional adjustment for lumbar spine BMD and serum levels related to Ca metabolism (iPTH, Ca, and P), higher BMD was associated with a lower risk of vitamin D insufficiency. Furthermore, elevated serum iPTH and lower serum Ca levels were significantly associated with both insufficiency and deficiency.

**Table 4:** Relative risk ratios (RRRs) for factors associated with vitamin D insufficiency and deficiency, compared with normal vitamin D status, in the fourth survey (2015–2016)

	VD insufficiency		VD defficiency	
Outcome: VD status (normal 0, insufficiency 1, deficiency 2) at the fourth survey	Model 1 Adjusted RRR (95% CI)	Model 2 Adjutsted RRR (95% CI)	Model 1 Adjusted RRR (95% CI)	Model 2 Adjutsted RRR (95% CI)
Number of subjects (total *N*= 1,902)	1,043		411	
Associated factors				
Sex (men 0, women1)	3.54 (2.71-4.65)***	3.16 (2.32-4.30)***	9.67 (6.54-14.3)***	10.0 (6.94-16.9)***
Age (+1 years)	0.99 (0.98-1.00)	0.99 (0.98-0.999)*	0.97 (0.95-0.98)***	0.96 (0.95-0.97)***
BMI (+1kg/m^2^)	1.05 (1.01-1.08)*	1.06 (1.02-1.11)**	1.05 (1.00-1.09)*	1.05 (1.00-1.10)*
Residential area (0: mountainous area, 1: coastal area)	0.59 (0.46-0.76)***	0.55 (0.43-0.71)***	0.40 (0.30-0.55)***	0.35 (0.25-0.48)***
Season of blood sampling (0: autumn, 1: winter)	2.07 (1.56-2.75)***	2.08 (1.56-2.77)***	2.79 (1.99-3.90)***	2.79 (1.98-3.95)***
Current smoking habit, yes	1.50 (1.01-2.23)*	1.56 (1.04-2.34)*	1.78 (1.03-3.06)*	1.90 (1.08-3.34)*
Current alcohol drinking, yes	0.69 (0.53-0.90)**	0.70 (0.53-0.91)**	0.56 (0.40-0.78)**	0.56 (0.40-0.79)**
Additional associated factors				
BMD at L2-4 (+1 g/cm^2^)		0.42 (0.22-0.80)**		0.42 (0.17-1.01)
Serum values of iPTH (+1 pg/mL)		1.01 (1.00-1.02)*		1.03 (1.02-1.04)***
Serum values of Ca (+1 mg/dL)		0.61 (0.44-0.85)**		0.21 (0.13-0.31)***
Serum values of P (+1 mg/dL)		1.11 (0.87-1.42)		0.85 (0/61-1.17)

*VD* vitamin D, *RRR* ralative risk ratio, *95% CI* 95% confidence interval, *BMI* body mass index, *BMD* bone mineral density, *L2-4* lumbar spine L2-L4, *iPTH* intact parathyroid hormone, *Ca* calcium, *P* phosphate

*p < 0.05, **p < 0.01, ***p < 0.001

## Discussion

This study analysed trends in the prevalence of hypovitaminosis D over a 10-year period in the general Japanese population. The results demonstrated a significant decrease in the prevalence of vitamin D deficiency from the baseline survey (2005–2007) to the fourth survey (2015–2016). Mean serum vitamin D levels increased, while the proportion of individuals with vitamin D deficiency significantly declined across all age groups and in both sexes.

The prevalence of vitamin D deficiency was significantly lower in the fourth survey than in the baseline survey in the initial multinominal regression model after ajudstment for sex, age, residential area, sempling season, and lifestyle factors, such as smoking and drinking. However, this difference was no longer significant after adjustment for serum Ca, P, iPTH, and lumbar spine BMD in addiiotn to the factors using the initial model. Nevertheless, the mean serum 25D level was significantly higher in the fourth survey, and the overall prevalence of deficiency remained lower, suggesting that vitamin D status has improved in the population over the decade.

Regarding recent trends in vitamin D insufficiency and deficiency in populations, Cui et al. reported, through a systematic review and meta-analysis of 308 peer-reviewed articles involving 7.9 million participants from 81 countries and WHO regions, that the global prevalence of serum 25D levels < 30 nmol/L (12 ng/mL) was approximately 15.7%, while levels < 50 nmol/L (20 ng/mL) reached approximately 47.9% [17]. This indicates that nearly half of the global population may be at risk of vitamin D insufficiency, which is associated with various health issues including osteoporosis and bone disorders. The study also indicated that the Eastern Mediterranean region experiences particularly high rates of vitamin D deficiency.

In the United States, based on data from 71,685 participants in the National Health and Nutrition Examination Survey, Cui et al. reported weighted prevalences of severe (< 25 nmol/L, 10 ng/mL) and moderate vitamin D deficiency (25–50 nmol/L, 10–20 ng/mL) of 2.6% and 22.0%, respectively. The prevalences of vitamin D insufficiency (50–75 nmol/L, 20–30 ng/mL) and sufficiency (> 75 nmol/L, 30 ng/mL) were 40.9% and 34.5%, respectively [18]. In Europe, Cashman et al. reported that among 55,844 individuals, the prevalence of vitamin D deficiency (serum 25D < 50 nmol/L) was 40.4% [19].

In the present study, the prevalences of vitamin D deficiency (serum 25D < 50 nmol/L) and vitamin D insufficiency (≥ 50 nmol/L and < 75 nmol/L) were approximately 21.6% and 54.8%, respectively, in the most recent (fourth) survey. Compared with data from the United States and Europe, the prevalence of vitamin D deficiency among ROAD study participants was lower than that in Europe and comparable to that in the United States. However, Miyamoto et al. reported that 98% of Japanese individuals fell into the category of hypovitaminosis D, using a fully automated liquid chromatography-tandem mass spectrometry method to measure serum 25D [20]. Therefore, it remains possible that the prevalence of vitamin D insufficiency in Japan is still high.

To our knowledge, there have been no previous studies that longitudinally followed the same general population to assess changes in vitamin D status over time. In the present study, during a 10-year observation period, the prevalence of hypovitaminosis D significantly decreased across all age groups and both sexes, accompanied by a significant increase in serum 25D levels. We previously reported trends in the prevalence of osteoporosis in the ROAD study; although the prevalence of osteoporosis in the femoral neck did not significantly decrease, that of L2–4 significantly declined in both men and women. In addition, the prevalence of osteoporosis in either the L2–4 or femoral neck among women aged ≥ 70 years was significantly lower in the fourth survey than in the baseline survey [21]. The observed increase in 25D levels in the present study may have contributed to the reduced prevalence of osteoporosis.

Interestingly, in our regression analysis, the significant decrease in vitamin D deficiency observed in the fourth survey compared to the baseline survey was no longer evident after adjusting for markers of mineral metabolism, such as Ca, P, and iPTH, along with demographic and lifestyle factors. This attenuation suggests that the improvement in vitamin D status over the decade may have led to favourable changes in mineral metabolism, particularly increased serum Ca and decreased iPTH levels, which in turn mediated the association. Given the known physiological interplay, it is plausible that improved vitamin D levels helped stabilise Ca–P homeostasis and parathyroid function, thereby diminishing the statistical association when these markers were included in the model.

The favourable change in the prevalence of hypovitaminosis D observed in this study can be attributed to three potential causes. The first is nutrition. The National Nutrition and Health Survey in Japan showed that the mean vitamin D intake among Japanese people slightly increased from 7.2 μg/day in 2005 [22] to 7.4 μg/day in 2015 [23], which is not a significant increase over the past decade. Regarding sources of vitamin D, fish intake—a primary source—decreased from 92.0 g/day to 74.6 g/day, whereas mushroom intake, another important source, increased from 7.0 g/day to 8.0 g/day. Although these changes are not striking, it is possible that the growing awareness of consuming vitamin D-rich foods over the past decade has contributed to the improvement in vitamin D status. Furthermore, data from the National Livelihood Survey, conducted every 3 years in Japan, indicate that the proportion of individuals consuming health supplements increased from approximately 20% in 2004 [24] to approximately 30% in 2016 [25], although there is no vitamin D-specific survey available. This suggests an increasing trend in the use of supplements, including vitamin D supplements often combined with calcium, which may have contributed to the improved nutritional status among users.

The second possible reason for the recent decrease in the prevalence of osteoporosis is the measures taken by governments and academic societies to prevent osteoporosis. In 1995, the Ministry of Health, Labour and Welfare recommended that all communities start BMD screening for middle-aged and elderly female residents. Although the proportion of women who participated in screening was not high, these examinations have increased the awareness of osteoporosis among women in various communities. Moreover, the Japan Osteoporosis Foundation and Japan Osteoporosis Society, established in 1991 and 1999, respectively, have been conducting public education campaigns using various advertising platforms to inform the general population about osteoporosis and its risk factors. The publicity efforts of academia, governments, and medical staff related to osteoporosis may contribute to a decrease in osteoporosis and, subsequently, the levels of 25D.

Finally, participation in previous BMD examinations in ROAD surveys might have been effective. The fourth ROAD study had three follow-up visits and a total of four survey exercises. The frequency of surveys within 10 years may have affected the results of the fourth survey. Although the ROAD study was an observational study and no intervention was applied to the participants, after the completion of each examination survey, the results were explained to the participants with medical advice. These experiences might have influenced the results of the fourth survey. In this case, the prevalence of hypervitaminosis D in the fourth survey may have been underestimated. However, this could also be interpreted as a bias resulting from increased awareness of osteoporosis owing to participation in the ROAD examination. It can be argued that enhanced knowledge about osteoporosis through examinations may have contributed to reducing the prevalence of vitamin D deficiency.

To identify the factors associated with hypovitaminosis D, we performed an analysis using data from the fourth survey. In the fully adjusted analysis, the risk of vitamin D deficiency was significantly higher among participants who were female, younger, and current smokers and had elevated serum iPTH levels than their counterparts. Notably, the BMI and BMD of the lumbar spine L2–4 were no longer significantly associated with vitamin D deficiency. Conversely, residing in coastal areas and current drinking habit were associated with a decreased risk of vitamin D insufficiency and deficiency. These findings were consistent with those of the baseline survey, which also identified younger age, female sex, residence in mountainous areas, smoking, and higher serum iPTH levels as factors associated with an increased risk of hypovitaminosis D [9].

Of particular concern was the consistently reported significantly higher risk of hypovitaminosis D in young women across all surveys, which warrants further investigation. Although recent studies on osteoporosis have reported a decline in the prevalence of lumbar spine osteoporosis [21] and improvements in vitamin D levels in the present study, the higher prevalence of hypovitaminosis D among younger individuals compared with older age groups is alarming. This trend suggests a potential future increase in osteoporosis prevalence, raising concerns about long-term skeletal health and the broader implications for public health. In the fourth ROAD Survey, the mean (SD) 25D levels based on age group among women were as follows: < 40 years, 20.1 (5.2); in their 40 s, 20.3 (5.6); in their 50 s, 22.4 (5.5); in their 60 s, 24.5 (6.1); in their 70 s, 24.4 (6.1); and > 80 years, 23.2 (6.1). These results indicate that the levels in women in their 30 s–50 s are lower compared with those aged > 60 years. These results highlight the urgent need to increase vitamin D levels in women before menopause to ensure future bone health. The academic community, osteoporosis societies, and government authorities should raise awareness about the increasing prevalence of vitamin D deficiency in young women and take more proactive measures to promote osteoporosis prevention.

This study has some limitations. First, although the study involved a large sample size, it was limited to specific regions of Japan, which may have affected the generalisability of the findings to the entire Japanese population. To address this issue, we compared anthropometric measurements, such as BMI, between the present study participants and the general Japanese population. The values of the 1,690 participants in the baseline study compared with those of the general population were obtained from the 2005 report of the National Health and Nutrition Survey conducted by the Ministry of Health, Labour and Welfare, Japan [22]. The mean BMI (SD) values of men aged 40, 50, 60, and ≥ 70 years, as reported in the 2005 National Health and Nutrition Survey, were 23.99 (3.27), 23.74 (3.07), 23.75 (2.94), and 23.26 (3.07), respectively, and those of women were 22.44 (3.49), 23.06 (3.37), 23.54 (3.66), and 23.04 (3.65), respectively. In the present study, the mean BMI values for men in identical age strata as that in the baseline study were 24.50 (4.46), 23.58 (2.91), 23.76 (3.22), and 22.58 (2.91), respectively, and those for women were 21.95 (4.08), 23.00 (3.24), 23.28 (3.16), and 22.88 (3.62), respectively. No significant differences were identified between our participants from the baseline study and the general Japanese population, except for male participants aged ≥ 70 years (*p* < 0.01). We further compared the mean BMI values of men aged 70 − 74, 75 − 79, and ≥ 80 years with those of the general Japanese population. The mean BMI (SD) values of men aged 70 − 74, 75 − 79, and ≥ 80 years, as reported in the 2005 National Health and Nutrition Survey, were 23.68 (3.18), 23.31 (3.04), and 22.27 (2.64), respectively, and those in the present study were 23.02 (2.90), 22.14 (2.81), and 22.56 (3.03), respectively, which suggests that only male participants aged 75 − 79 years in the present study (from the baseline ROAD study alone) had significantly smaller body build than the overall Japanese population in the same age stratum. A similar comparison was performed for the BMI values of 1,906 participants (636 men, 1,270 women) in the fourth ROAD survey and the values obtained from the 2015 report of the National Health and Nutrition Survey [23] when the fourth ROAD survey was performed. No significant differences were identified between our survey participants and the general Japanese population (both men and women aged 40 s, 50 s, 60 s, and ≥ 70 years). In summary, the significant difference in BMI among men aged 75–79 years in the ROAD baseline study indicates that this population is not representative of the Japanese population. Therefore, this difference should be considered when evaluating potential risk factors for osteoporosis in men in the baseline ROAD survey.

Additionally, seasonal variations in blood sample collection may have influenced serum 25D levels, because vitamin D levels can fluctuate with changes in sunlight exposure throughout the year. In the baseline survey, examinations were conducted from October to January of the following year, whereas in the fourth survey, examinations were conducted from October to December. To minimise the impact of seasonal variations, when the same participants took part again, we checked the date of their previous examination and scheduled a new examination as close as possible to that date. However, it is unclear whether we were able to completely eliminate seasonal effects.

In addition, data on direct measures of sun exposure—such as duration or intensity—were not collected in either the baseline or the fourth survey in these areas. Given that ultraviolet B (UVB) radiation is a key determinant of vitamin D synthesis, this absence precludes adjustment for changes in sun exposure over time. To better contextualise the observed trends in serum vitamin D levels, we reviewed official solar exposure data for Wakayama Prefecture from the Japan Meteorological Agency (JMA) for the years 2005 and 2015. In 2005, the annual total sunshine duration in Wakayama was approximately 2,071.2 h. The mean daily global solar radiation (all-sky insolation) was about 13.4 MJ/m^2^/day for the year. In contrast, in 2015, the annual sunshine duration was slightly lower, at approximately 1,964.8 h. Despite the decrease in sunshine hours, the mean daily global solar radiation was slightly higher at about 15.2 MJ/m^2^/day. (Data source: Japan Meteorological Agency (JMA), Historical Climate Data for Wakayama Station.) These findings suggest that while there was a modest decline in total sunshine duration over the 10-year period, the intensity of solar radiation slightly increased, resulting in no substantial change in overall potential for cutaneous vitamin D synthesis. Therefore, it is unlikely that changes in solar exposure alone explain the observed reduction in vitamin D insufficiency. Other factors, such as improvements in osteoporosis management, increased awareness of bone health, or changes in dietary or supplement intake, may have contributed to this trend.

In conclusion, this study provides evidence of a significant decrease in the prevalence of vitamin D deficiency over a 10-year period in a Japanese cohort. Improvement in the vitamin D status may contribute to a reduction in osteoporosis and associated fractures, highlighting the importance of monitoring and maintaining adequate vitamin D levels in the general population. However, concerns regarding the increasing prevalence of hypovitaminosis D among young women have also been highlighted. Continued efforts to understand and address the determinants of hypovitaminosis D are crucial for public health in Japan.

## Data Availability

Not applicable.
