# Correction: Trends in the prevalence of hypovitaminosis D over a 10-year period in Japan: the research on osteoarthritis/osteoporosis against disability study 2005–2015

**DOI:** 10.1007/s11657-025-01608-2

**Published:** 2025-09-19

**Authors:** Noriko Yoshimura, Toshiko Iidaka, Chiaki Horii, Gaku Tanegashima, Shigeyuki Muraki, Hiroyuki Oka, Hiroshi Kawaguchi, Toru Akune, Kozo Nakamura, Sakae Tanaka

**Affiliations:** 1 Department of Prevention Medicine for Locomotive Organ Disorders, 22nd Century Medical and Research Center, The University of Tokyo, Tokyo, 113-8655 Japan; 2 Department of Orthopaedic Surgery, Sensory and Motor System Medicine, Graduate School of Medicine, The University of Tokyo, Tokyo, 113-8655 Japan; 3 Division of Musculoskeletal AI System Development, Faculty of Medicine, The University of Tokyo, Tokyo, 113-8655 Japan; 4 Nodogaya Hospital, Chiba, 277-0084 Japan; 5 National Rehabilitation Center for Persons With Disabilities, Saitama, 359-0042 Japan; 6 Towa Hospital, Tokyo, 120-0003 Japan

**Correction: Archives of Osteoporosis (2025) 20:117**

10.1007/s11657-025-01601-9

The original online version of this article was revised:

In the originally published version of this article:

“Trends in the prevalence of hypovitaminosis D over a 10-year period in Japan: the research on osteoarthritis/osteoporosis against disability study 2005–2015” (DOI: 10.1007/s11657-025-01601-9),

several comments intended as author instructions for table formatting were inadvertently included in the published PDF. These comments, highlighted in yellow during the proofreading stage (e.g., “Please correct the table formatting…”; “Please adjust the background shading…”), appeared before Tables 1, 2, 3, and 4.

The comments were purely for internal proofreading purposes and should not have appeared in the final published version. The PDF has now been corrected to remove these comments. The HTML version of the article was unaffected.

The original article has been corrected.

